# Reliability of cognitive tests of ELSA-Brasil, the brazilian longitudinal study of adult health

**DOI:** 10.1590/S1980-57642013DN74000003

**Published:** 2013

**Authors:** Juliana Alves Batista, Luana Giatti, Sandhi Maria Barreto, Ana Roscoe Papini Galery, Valéria Maria de Azeredo Passos

**Affiliations:** 1 Enfermeira, Mestre em Ciências da Saúde pelo Programa de Pós-graduação em Ciências da Saúde, Universidade Federal de Minas Gerais, Belo Horizonte MG, Brasil.; 2 Médica, Doutora em Epidemiologia, Professora Adjunta da Escola de Nutrição, Universidade Federal de Ouro Preto, Ouro Preto MG, Brasil.; 3 Médica, Doutora em Epidemiologia, Professora Titular do Departamento de Medicina Preventiva e Social, Faculdade de Medicina, Universidade Federal de Minas Gerais, Belo Horizonte MG, Brasil. Coordenadora do ELSA-Brasil.; 4 Estudante de Iniciação Científica, Faculdade de Enfermagem, Universidade Federal de Minas Gerais, Belo Horizonte MG, Brasil.; 5 Médica, Especialista em Geriatria, Doutora em Medicina, Professora Associada do Departamento de Clínica Médica, Faculdade de Medicina, Universidade Federal de Minas Gerais. Vice-Coordenadora do Programa de Pós Graduação em Ciências da Saúde, Universidade Federal de Minas Gerais, Belo Horizonte MG, Brasil.

**Keywords:** cognitive assessment, reliability, cohort studies

## Abstract

Cognitive function evaluation entails the use of neuropsychological tests, applied exclusively or in sequence. The results of these tests may be influenced by factors related to the environment, the interviewer or the interviewee.

**OBJECTIVES:**

We examined the test-retest reliability of some tests of the Brazilian version from the Consortium to Establish a Registry for Alzheimer's disease.

**METHODS:**

The ELSA-Brasil is a multicentre study of civil servants (35-74 years of age) from public institutions across six Brazilian States. The same tests were applied, in different order of appearance, by the same trained and certified interviewer, with an approximate 20-day interval, to 160 adults (51% men, mean age 52 years). The Intraclass Correlation Coefficient (ICC) was used to assess the reliability of the measures; and a dispersion graph was used to examine the patterns of agreement between them.

**RESULTS:**

We observed higher retest scores in all tests as well as a shorter test completion time for the Trail Making Test B. ICC values for each test were as following: Word List Learning Test (0.56), Word Recall (0.50), Word Recognition (0.35), Phonemic Verbal Fluency Test (VFT, 0.61), Semantic VFT (0.53) and Trail B (0.91). The Bland-Altman plot showed better correlation of executive function (VFT and Trail B) than of memory tests.

**CONCLUSIONS:**

Better performance in retest may reflect a learning effect, and suggest that retest should be repeated using alternate forms or after longer periods. In this sample of adults with high schooling level, reliability was only moderate for memory tests whereas the measurement of executive function proved more reliable.

## Abstract (pt)

A avaliação da cognição utiliza testes neuropsicológicos, aplicados isoladamente ou em sequência. Os resultados destes testes podem sofrer influências relacionadas ao ambiente, ao entrevistador ou ao entrevistado.

**OBJETIVOS:**

Investigar a confiabilidade teste-reteste de alguns testes da versão brasileira do Consortium to Establish a Registry for Alzheimer's disease.

**MÉTODOS:**

O ELSA-Brasil é uma coorte multicêntrica de servidores (35-74 anos) de instituições públicas de seis estados. Os mesmos testes foram aplicados, em ordens distintas, pelo mesmo entrevistador treinado e certificado, com intervalo aproximado de 20 dias, entre 160 participantes (51% homens, média de idade de 52 anos). O Coeficiente de Correlação Intraclasse (CCI) foi usado na verificação da confiabilidade das medidas, e um gráfico de dispersão evidenciou padrões de concordância entre teste e reteste.

**RESULTADOS:**

Foram observados maiores escores em todos os retestes, assim como menor tempo para completar o Teste de Trilhas B (Trilhas B). Os valores de CCI para cada teste foram: memória imediata de palavras (0,56), evocação (0,50), reconhecimento (0,35), Teste de Fluência Verbal fonêmica (TFV, 0,61), TFV semântica (0,53) e Trilhas B (0,91). O gráfico de Bland-Altman mostrou melhor correlação dos testes de função executiva (TFV e Trilhas B) que dos testes de memória.

**CONCLUSÕES:**

O melhor desempenho nos retestes parece refletir efeito de aprendizado e sugere que o reteste seja aplicado após períodos mais longos ou com formas alternativas dos testes. Nesta população com predominância de adultos jovens e de alta escolaridade, a confiabilidade foi moderada para testes de memória e maior para testes de função executiva.

## INTRODUCTION

Human cognition refers to the acquirement of knowledge by means of a complex interaction of the neural networks, which form the mental processes connected to thinking, perception, memory and premeditated action. The study of cognitive functions includes clinical and neuropsychological evaluations. Neuropsychological evaluation includes the use of tests, applied exclusively or in sequence, to assess functional and intellectual abilities. These tests attempt to capture and describe complex phenomena in a standardized manner, so they can be analysed in clinical and epidemiologic studies.^1^

In scientific studies, evidence must be based on valid results, with no methodological errors in the conception, design and implementation of the study, or in the process of data analysis. The application of cognitive tests may be influenced by many factors, which can interfere in their results. There are factors that can be minimized by controlling the quality of the study. Training and certification can prevent sources of variability associated with the examiner, such as: intonation while giving the instructions, the ability to create a professional and friendly environment, experience with the test, following the correct technique, giving neutral answers to patients' questions, repeating the questions and not interpreting them etc. The test orientations should be able to prevent or attenuate some aspects related to the patient, such as fatigue, sleep deprivation, mood and readiness to take the test. Nonetheless, there are sources of variability associated with the test itself. These may be assessed by the test's validity and reliability. The latter indicates the extent to which the test can obtain the same results when reapplied, maintaining the same original conditions.^2^

In studies that examine the reliability of cognitive assessments, the test is considered precise when the results obtained upon its reapplication are consistent with the results from the first application. In the retests, one strives to maintain the same application conditions, considering the variables that interfere with performance, such as: environment, privacy, luminosity, the examiner's and the participant's situation. The time gap between the test and the retest is also an important factor to be considered. Long periods are associated with changes, such as alterations in cognitive capacity. Short periods, on the other hand, increase the probability of the learning effect, whereby participants remember their answers from the first test and simply repeat them in the retest.^3^

A battery of cognitive tests was used in the Longitudinal Study of Adult Health (ELSA-Brasil), which involves a cohort of 15,105 public civil servants. The object of the study is to investigate the incidence and progression of non-communicable chronic diseases, and to examine the biological, behavioural, environmental, occupational, psychological and social factors associated with these diseases and their complications, in an attempt to build a causal model which reflects their inter-relations.^4^ This battery of tests employs some of the neuropsychological tests from the Consortium to Establish a Registry for Alzheimer's disease (CERAD).^5^ The CERAD cognitive test battery, widely used in clinical and epidemiological studies, is described as having many advantages, such as: detecting dementia in its initial phase, allowing comparison of results from different groups, and offering good test-retest reproducibility and substantial interrater reliabilities.^6^ This battery was adapted and validated for use in Brazil in 1998, but its reliability has not yet been tested in this country. However, evaluation of performance on cognition tests show that younger age and higher schooling levels are associated with better performance.^7-9^

The reliability of a test is highly influenced by the characteristics of the population that takes it. 1 Therefore, the objective of this study was to assess the reliability, by test and retest, of those cognitive tests applied in the ELSA-Brasil population and, furthermore, to investigate their reliability according to age, sex and schooling.

## METHODS

**Participants.** The ELSA- Brasil is an ongoing multicentre study of volunteer adults (35 to 74 years of age) from public teaching and research institutions across six states in Brazil: Bahia, Espírito Santo, Minas Gerais, Rio de Janeiro, Rio Grande do Sul and São Paulo. This test-retest reliability study was performed on a convenience sample formed by 160 participants, selected according to pre-established quotas for sex and age groups (35-44, 45-64 and 65+ years), from one ELSA research centre in Minas Gerais. ELSA-Brasil was approved by the Research Ethics Committee of each of the institutions, including the Research Ethics Committee of the Federal University of Minas Gerais (COEP UFMG), and all participants signed an informed consent form (ETIC 186/06).^4^

**Instruments and procedures.** The battery of cognitive tests was applied twice, with an interval of 14-27 days (mean =20±3 days). The memory tests (immediate recall, evocation and recognition) compromised a list of ten unrelated words printed in large letters on cards, with the words shown every 2 seconds and presented in a different order on each of the three learning trials, with immediate recall. After a 5 minutes' delay, retention and recollection were tested by a free recall and by the recognition of ten previous words that were intermixed with ten distractor words. Verbal fluency tests (VFT) consisted of asking participants to say in one minute as many words as possible related to a specific category of animals (semantic test) or beginning with the letter F (phonemic test). The Trail Making Test B (Trail B), part a, was used to train for Trail B, part b, with the time taken to complete the task computed only for part b. The participant was instructed to draw lines connecting letters and numbers in an order that alternates between increasing numeric value and alphabetic order (1,A, 2,B, 3,C, etc.).The participant had to draw as quickly as possible, without lifting the pencil tip from the page. Supervisors were instructed to point out the errors. The test score was the total time to complete the condition, including the time necessary to correct errors.^5^

The same tests were applied, albeit in a different order, between 22/02/2010 and 03/12/2010 by the same previously-trained and certified interviewer, in a quiet environment, with good lighting and low levels of noise or other distracting stimulations. The order of the tests was arranged in such a manner that there was always a diverting test, category/phonemic or phonemic/category VFT, between the word memory test and the recall and recognition tests. The Trail B was always the first or the last test to be performed. The tests were recorded and later revised. VFT scores were defined by previously-trained and certified supervisors from the ELSA-Brasil research centres. A high level of agreement was observed between each of the six centres and the reference standard.^10^

**Statistical analysis.** The Epiinfo^®^ 3.5.3Program, 10 was used for the double data entry, and the STATA^®^ Program, 12 for the statistical analysis.

Descriptive analysis of the tests and retests was generated by means of the average and the range of variation in first and second application. As homogeneity was found only for Trail B data (Bartlett Test <0.05), the Mann Whitney test was used to compare the average time between test and retest.

The Intraclass Correlation Coefficient (ICC) was used as the main measure for estimating reliability, since this test assesses the total variability caused by differences between individuals. The ICC reliability test was done according to the characteristics of the participants: sex, age range (35-59 and 60-74 years-old) and educational level (uncompleted high school, completed high school, University).

Reliability, according to ICC values, was classified as poor when equal to zero; slight – from 0.01 to 0.2; fair – from 0.21 to 0.4; moderate – from 0.41 to 0.6; substantial – from 0.61 to 0.8; almost perfect – from 0.81 to 0.9.13

In order to compare our results with other studies, Pearson Correlation Coefficients were also estimated for memory tests and VFT. The Spearman Coefficient was estimated to compare the Trail B test. The Pearson coefficient measures the degree to which a paired group of observations in a diagram approaches a situation where each point is located precisely over the straight line, which means the absence of difference between two observations. Dispersion graphics were used to evaluate the pattern and distribution of scores.

## RESULTS

The study sample had the same sex and age distribution as the ELSA cohort and compromised 81 (50.6%) men and 79 (49.4%) women, 121 (75.6%) adults (35-59 years old) and 39 (24.4%) elderly (60-74 years old). A higher schooling level (10.6% had uncompleted high school, 28.8% completed high school and 60.6% had a University degree) than the participants of the cohort was observed.^4^

In addition, higher retest scores on the word memory, recall, semantic and phonemic VFT tests and a shorter retest time to perform the Trail B (Table 1), were also observed. The ICC varied from 0.35, for the recognition test, to 0.91, for the Trail B, which means that the capacity of the different tests to discriminate between individuals ranged from between moderate and almost perfect, respectively. All the tests presented a positive correlation, with statistically significant values, revealing that the retest scores tended to increase linearly in relation to the test scores (Table 2).

**Table 1:** Score distribution of cognitive tests and retests among 160 participants of ELSA-Brasil.

Measures	Tests					
	Word memory	Recall	Recognition	VFT* (animals)	VFT (letter F)	TRAIL B (seconds)
Range - test	11-28	1-10	8-10	6-35	3-27	29-858
Range - retest	12-30	2-10	7-10	10-34	2-26	31-526
Average - test	21	7	10	19	13	90.0
Average - retest	23.5	8	10	20	14	81.5**
Difference	p<0.001	p <0.001	p=0.42	p<0.001	p<0.001	p=0.009

* VFT: Verbal Fluency Tests;

** Trail B mean execution time and Mann-Whitney test .

**Table 2:** Test-retest reliability of cognitive tests performed in 160 participants of ELSA-Brasil.

Test	ICC* (95% CI**)	Pearson coefficient***
Word memory	0.56 (0.33-0.79)	0.74
Recall	0.50 (0.17-0.83)	0.68
Recognition	0.35 (0.00-0.94)	0.40
VFT****(animals)	0.53 (0.33-0.74)	0.72
VFT (letter F)	0.61(0.41-0.81)	0.77
Trail making test B*****	0.91 (0.87-0.95)	0.76

* ICC: Intraclass Correlation Coefficient;

** CI: Confidence Interval;

*** p<0.0001;

**** VFT: Verbal Fluency Tests;

***** Spearmann coefficient.

**Figure 1:**
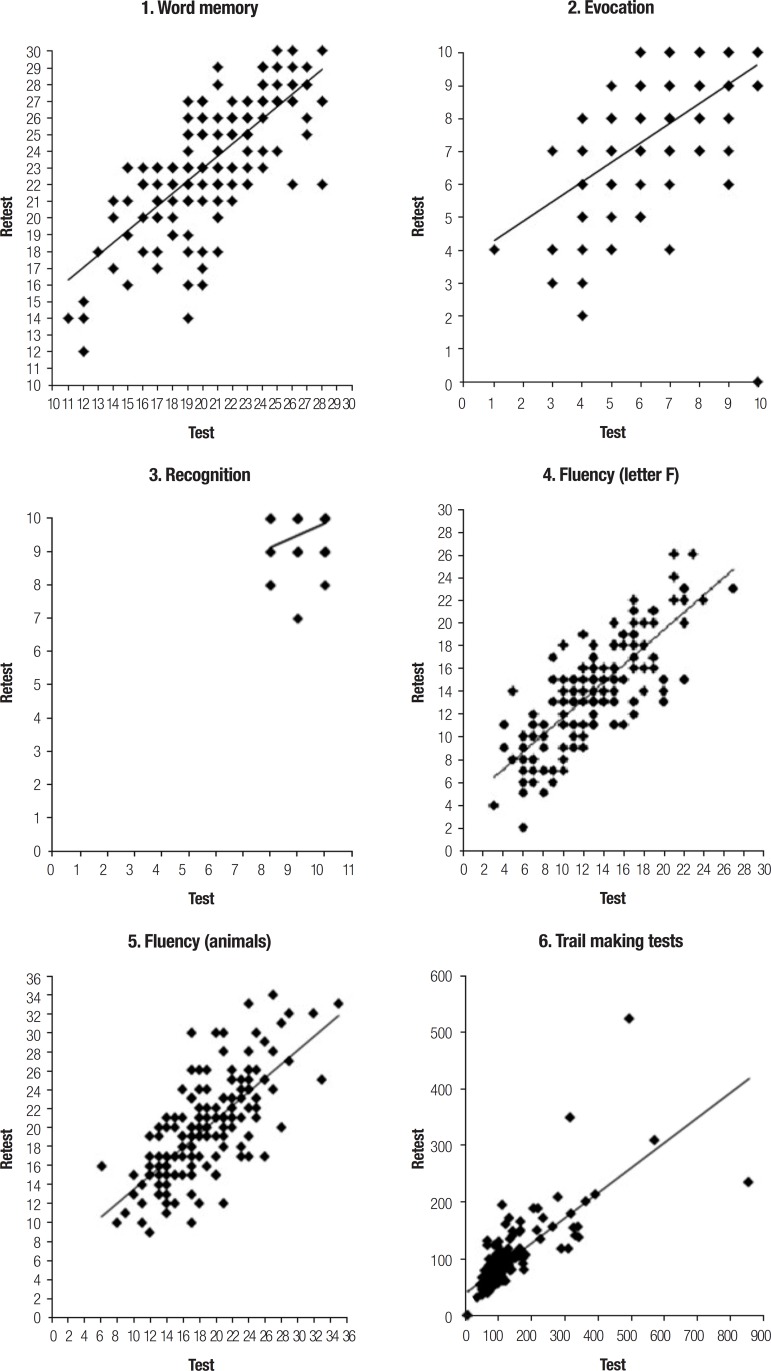
Test and retest dispersion graphs for battery of cognitive function tests among 160 participants of ELSA-Brasil.

Figure 1 depicts the dispersion graphs corresponding to Pearson coefficient values for the cognitive tests. The inclination of the line deviating from 45º shows the memory tests and VFT retest scores were higher than the test scores, while the opposite occurred with the Trail B. The recall test graph shows a higher dispersion of values. In the recognition tests, the presence of scores close to ten, the maximum limit in the test, is notable.

No influence of sex, age or schooling on reliability was found when variability of all test scores were analysed according to these variables, using stratified ICC values and their confidence intervals (Table 3).

**Table 3:** Intraclass Correlation Coefficient of cognitive function tests in ELSA-Brasil, by sex, age and schooling.

Variable		Word memory	Recall	Recognition	VFT*(animals)	VFT (letter F)	Trail B
Sex	Female	0.63 (0.39-0.86)	0.52 (0.16-0.88)	0.61 (0.00-1.22)	0.54 (0.30-0.78)	0.53 (0.28-0.78)	0.88 (0.80-0.96)
	Male	0.45 (0.16-0.74)	0.50 (0.15-0.85)	0.30 (0.00-0.89)	0.53 (0.29-0.77)	0.64 (0.42-0.86)	0.97 (0.96-0.99)
Age group (years)	35-59	0.56 (0.31-0.80)	0.49 (0.15-0.84)	0.26 (0.01-0.78)	0.57 (0.36-0.78)	0.61 (0.41-0.82)	0.90 (0.85-0.95)
	60-74	0.56 (0.24-0.88)	0.47 (0.07-0.86)	0.50 (0.00-1.19)	0.23 (0.00-0.62)	0.78 (0.58-0.97)	0.76 (0.28-1.24)
Schooling	Uncompleted High School	0.27 (0.00-0.88)	0.07 (0.00-0.67)	0.64 (0.01-1.36)	0.07 (0.00-0.89)	0.93 (0.70-1.06)	0.72 (0.24-1.20)
	Completed High School	0.71 (0.47-0.94)	0.56 (0.18-0.94)	0.30 (0.00-0.92)	0.41 (0.07-0.75)	0.49 (0.17-0.81)	0.89 (0.77-1.00)
	University	0.49 (0.22-0.76)	0.48 (0.11-0.84)	0.32 (0.00-0.89)	0.45 (0.22-0.69)	0.57 (0.33-0.80)	0.72 (0.24-1.20)

* VFT: Verbal Fluency Tests;

** Trail B: Trail Making Test B.

## DISCUSSION

The knowledge of reliability measures can be of clinical use and are very important for epidemiological studies, especially those with populations from countries with different schooling, culture and language than those where the tests were developed. Few publications have evaluated the reliability of the tests used in the present study.^5,14-16^ Although we used the adaption of the CERAD protocol proposed for Brazil,^17^ it is important to know the reliability of cognitive tests for the ELSA-Brasil population, as well as for any study which includes a study population other than the population originally investigated. In the CERAD study, the reliability was assessed among 610 individuals with or without Alzheimer's disease, with an average age of 68 years.^5^

In epidemiological studies, one rarely obtains the reproducibility level found in laboratory investigations, where it is easier to maintain identical evaluation conditions. In the present study, the word memory, recall and semantic VFT test revealed moderate reliability; the phonemic VFT, substantial reliability; and the Trail B almost perfect reliability. The higher reliability of the category VFT and Trail B suggests that they are more precise and less influenced by time of reapplication, since processing speed is common to both tests and is less affected by the test-retest effect.

In this study, it was decided to measure reliability using the ICC. The Pearson coefficient was used only for the sake of comparison with other studies that employed it, since the Pearson coefficient allows assessment of the correlation between variables, but not the difference between the evaluations. Our results are similar to the findings of studies carried out with other populations. Moderate reliability for the word memory test was found in a study from Korea^15^ and another study from the United States of America,^5^ which used samples of 20 and 278 people, respectively, aged under 50 years, and a one-month interval between test and retest. These same studies revealed substantial reliability for the recall test (Pearson coefficient=0.64). Lower reliability for the recognition test was also observed in the American study (Pearson=0.36), but not in the Korean investigation (Pearson=0.74).^15^ In our study, lower reliability for the recognition test may be explained by the ceiling effect, where the test values achieved by the sample are close to the maximum, reducing the variability between scores.

The better performance in the retests strongly suggests a learning effect, as observed in other studies.^18^ In the present study, we chose an interval of time similar to that adopted in other investigations, which ranged from two to four weeks. Longer periods between the tests could increase the probability of real changes in cognitive function, compromising the test reliability of the investigation; whilst shorter periods are more easily contaminated by the learning effect. As in other studies, in an attempt to avoid the learning effect, the retests were arranged in a different order. Despite these precautions, the influence of the learning effect may have contributed to decreasing the reliability of the tests.

Considering the Trail B showed almost perfect reliability, it may be useful when a short reapplication interval is necessary. High reliability for the Trail B test was also found in a German study, using a sample of 55 individuals, with a mean age of 46 years and, on average, 10 years of schooling.^16^

The studied tests presented the advantage of maintaining reliability regardless of sex, age and schooling. The test's capacity to be precise even when applied to different people should not, however, lead to the conclusion that these variables have no effect when assessing the validity of these tests. There is evidence that these variables interfere with the capacity to distinguish between cognitive levels.^9,19-20^

One limitation of this study is that it was conducted in one of the six ELSA research centers, as it was decided to reduce the variability of using different interviewers.

In conclusion, we observed moderate reliability for cognitive tests applied in adults, after a short interval averaging twenty days. The slight improvement in performance across all the retests, compared to the initial tests, suggests a learning effect. To avoid this effect, the ELSA-Brasil cognitive evaluation should use alternate equivalent versions of the test during study waves, estimated to be every three to four years, in order to reduce the influence of learning on prospective comparisons of cognitive tests in this Brazilian adult population.
